# Material heterogeneity of male genitalia reduces genital damage in a bushcricket during sperm removal behaviour

**DOI:** 10.1007/s00114-020-01706-w

**Published:** 2020-11-25

**Authors:** Yoko Matsumura, Mohsen Jafarpour, Steven A. Ramm, Klaus Reinhold, Stanislav N. Gorb, Hamed Rajabi

**Affiliations:** 1 Department of Functional Morphology and Biomechanics, Zoological Institute, Kiel University, Am Botanischen Garten 1–9, 24118 Kiel, Germany; 2 Department of Evolutionary Biology, Bielefeld University, Konsequenz 45, 33615 Bielefeld, Germany

**Keywords:** Confocal laser scanning microscopy, Finite element modelling, Cuticle, Durability, Genital coupling, Material heterogeneity

## Abstract

Sperm removal behaviour (SRB) is known in many animals, and male genital structures are often involved in the SRB, e.g. rubbing female genitalia vigorously. However, it remains unclear how those male genital structures function properly without severe genital damage during SRB. In the present study, we focused on the bushcricket *Metaplastes ornatus* and examined the biomechanics of male and female genital structures, involved in their SRB as a model case. During an initial phase of mating, males of this species thrust their subgenital plate with hook-like spurs and many microscopic spines into the female genital chamber. By moving the subgenital plate back-and-forth, males stimulate females, and this stimulation induces the ejection of sperm previously stored in females. We aimed to uncover the mechanics of the interaction between the subgenital plate and genital chamber during SRB. The genital morphology and its material composition were investigated using modern imaging and microscopy techniques. The obtained results showed a pronounced material heterogeneity in the subgenital plate and the genital chamber. The material heterogeneity was completely absent in that of a second bushcricket species, *Poecilimon veluchianus*, which does not exhibit SRB. Finite element simulations showed that the specific material heterogeneity can redistribute the stress in the subgenital plate of *M. ornatus* and, thereby, reduces stress concentration during SRB. This may explain why only a few examined males had a broken spur. We suggest that the observed structural features and material heterogeneity in *M. ornatus* are adaptations to their SRB.

**Supplementary Information:**

The online version contains supplementary material available at 10.1007/s00114-020-01706-w.

## Introduction

Sperm removal behaviour triggered by male stimulation (hereafter SRB) is an offensive male adaptation to achieve fertilisation success under sperm competition (Parker 1970; Simmons 2001). There are two types of SRB. In one of them, males stimulate female genitalia, which results in sperm ejection by females, and in the other one, males physically remove sperm stored in females (e.g. Waage 1979, 1986a, b; Ono et al. 1989; Yokoi 1990; von Helversen and von Helversen 1991; Haubruge et al. 1999; Kamimura 2000; Gallup et al. 2003; Matsumura et al. 2014). Many examples of SRB have been reported across several insect orders, including dragonflies and damselflies (e.g. Waage 1979, 1986a, b), an earwig (Kamimura 2000), a bushcricket and a tree cricket (Ono et al. 1989; von Helversen and von Helversen 1991), various beetles (e.g. Yokoi 1990; Gack and Peschke 1994; Haubruge et al. 1999) and possibly an angel insect (Matsumura et al. 2014). Given the widespread potential adaptive significance of such sperm removal in securing paternity success, it is not surprising that equivalent adaptations are also seen in other taxa (e.g. Naud et al. 2004; Wada et al. 2005; Galeotti et al. 2007; Calbacho-Rosa et al. 2013), and a similar function has been proposed for the human penis (Gallup et al. 2003).

From a male perspective, the removal of rival sperm from female sperm storage organs is always likely to be beneficial due to increased fertilisation success. On the other hand, sperm removal could be counter to female interests since females actively invest in maintaining sperm in female sperm storage organs, e.g. providing nutrition to sperm and protecting sperm against pathogens (Orr and Zuk 2012; Orr and Brennan 2015; Pascini and Martins 2017). Moreover, in one bushcricket species, a female had carried wounds in the reproductive tract, possibly inflicted by a male genital organ during the SRB (von Helversen and von Helversen 1991). Although it is not yet disentangled whether females eject sperm to minimise costs inflicted by SRB or whether SRB induces the ejaculation, considering these potential functions and costs of female sperm storage, SRB likely causes sexual conflict (Arnqvist and Rowe 2005). Therefore, it is reasonable to expect that intromittent organs used for SRB are specialised to stimulate or rake the sperm effectively and that females exhibit counterstrategies. Nevertheless, the mechanics of the interaction between male and female genitalia during SRB have not been addressed.

**Fig. 1:**
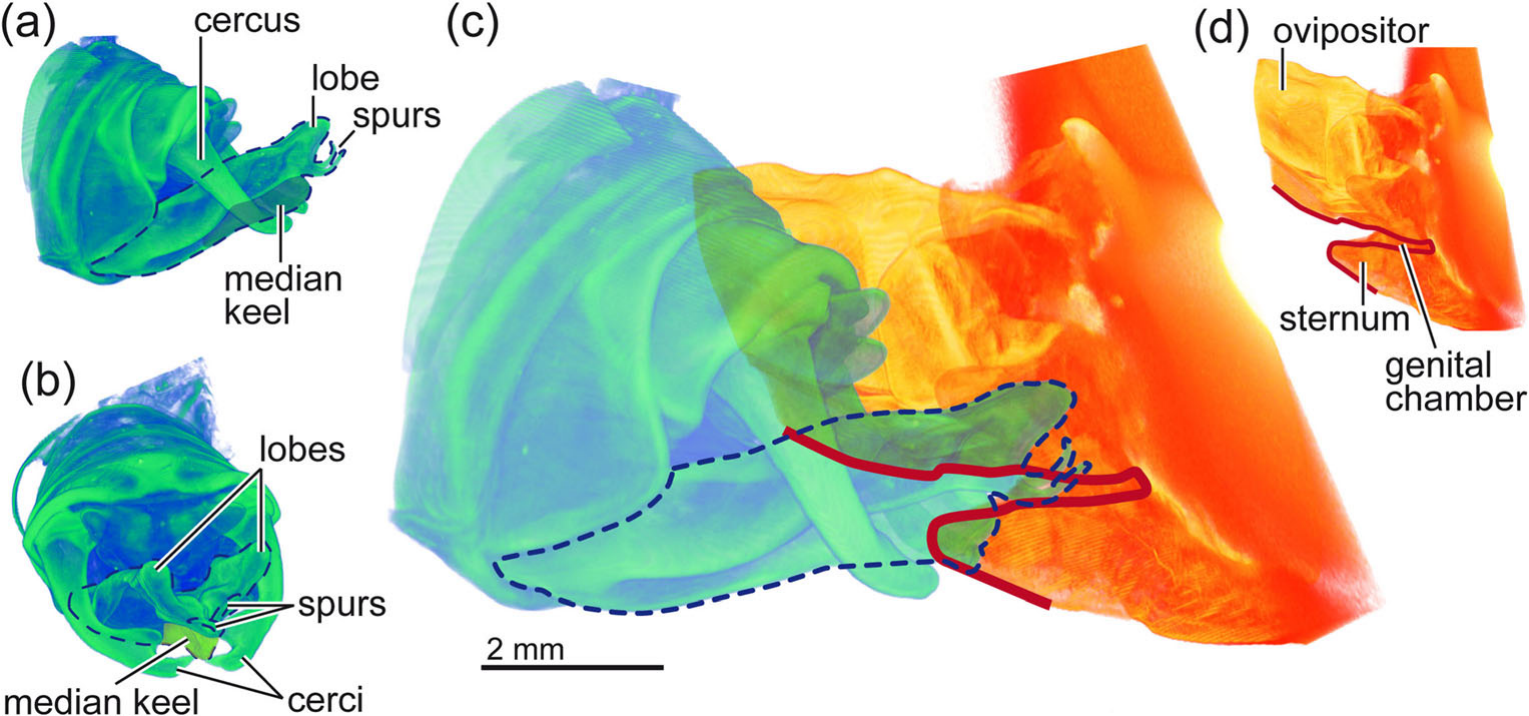
Genital coupling based on 3D scanned genital structures in *Metaplastes ornatus*. (**a**) A male postabdomen in the lateral view. (**b**) A male postabdomen in the caudal view. (**c**) Simulated genital coupling. The male part was coloured with bluish green, and female one was with yellowish orange. The outline of the genital chamber is depicted with the red line, and the outline of the subgenital plate is shown by the dashed blue line. (**d**) A part of female postabdomen in the lateral view

Here we use the bushcricket *Metaplastes ornatus* Ramme 1931 (Orthoptera, Tettigonoidea, Phaneropteridae) as a study species because it is highly suited to a biomechanical study of genital interactions during SRB. Males of this species thrust a part of the postabdomen—the subgenital plate—into the female genitalia prior to the transfer of their own ejaculate (von Helversen and von Helversen 1991). This stimulation causes the ejection of previously stored sperm by females, indicating that this part of the postabdomen functions solely for sperm removal. Males use their subgenital plate equipped with two rearwards-projecting lobes, a median keel composed of two sharp sclerotised spurs and pointed barbs covering the surface (Fig. 1) (von Helversen and von Helversen 1991). Although the morphology of the subgenital plate is highly variable among Ensifera (Ander 1970), these features are likely derived characters that presumably function in effecting or supporting the SRB. Males persistently thrust the subgenital plate into the female genital chamber and vigorously rub against the walls of the genital chamber with the subgenital plate (a video is available as a supplementary file in Foraita et al. 2017; see also von Helversen and von Helversen 1991). The number of thrusts is 685–834 on average when males copulate with non-virgin females, but it can exceed 2000 in one copulation (Foraita et al. 2017). Scars in the female chamber have also been reported as a consequence of this vigorous rubbing (von Helversen and von Helversen 1991). The sperm removal efficiency of the species is relatively high, about 85% (von Helversen and von Helversen 1991). As a result of the effectiveness of the SRB, realised sperm competition levels are likely weaker, which may, for example, explain the relatively small testes and negative testes allometry detected in this species (Winkler et al. 2019).

*M. ornatus* males need to avoid breakage of the subgenital plate despite their vigorous SRB to maintain its function. Moreover, females are not simply passive recipients of such behaviour. We observed, at least in captivity, that females reject males quite often during SRB (YM, SAR & KR pers. observ. 2019). In the current study, to unveil if there are any adaptive strategies to avoid genital damage in both sexes, we aimed to address the following questions: (1) how often the male and female genitalia get damage in field-caught bushcrickets and (2) are there any strategies to reduce genital damage that could be inflicted during SRB? For this purpose, first, we observed field-caught males and females to quantify the frequency and severity of genital injuries. We then used computed tomography and microscopy to investigate the morphology and material distributions of the relevant male and female genital structures, using the results from which to perform finite element simulations, to address how males avoid breakage of the delicate, paired hook-like spurs of the subgenital plate. As a comparison, we also analysed the material distribution from corresponding structures of *Poecilimon veluchianus veluchianus* Ramme, 1933 (Orthoptera, Tettigonoidea, Tettigoniidae), a bushcricket species which does not exhibit SRB. Based on our results, we discuss how *M. ornatus* males reduce damage to their genitalia during SRB.

## Materials and methods

### Collection and preparation of specimens

Specimens of *M. ornatus* and *P. v. veluchianus* were collected in Paleokastro, central Greece (Nomos Fthiothis), and further specimens of *M. ornatus* were collected a few kilometre north of the village of Vitoli, central Greece (Nomos Fthiothis), between 16 June 2019 and 26 June 2019. The specimens were first frozen in a conventional freezer and preserved in 70% ethanol for a few weeks before we conducted further experiments.

### Simulated genital coupling

Since it was challenging to fasten a male and female during the SRB in the field, we adopted a virtual genital coupling strategy. A male and female of *M. ornatus* were scanned using a SkyScan 1172 micro-computed tomography (microCT) scanner (Bruker micro-CT, Kontich, Belgium). For this purpose, the samples were dehydrated with an ascending series of ethanol to absolute ethanol, which was then substituted with 1,1,1,3,3,3-hexamethyldisilazane (HMDS, Carl Roth GmbH & Co. KG, Karlsruhe, Germany), and were dried in a fume hood. HMDS was used for drying the samples as an alternative method of critical point drying, which was first applied to insect specimens for scanning electron microscopy (Nation 1983). The current and source voltage were set at 250 μA and 40 kV in the male and 192 μA and 32 kV in the female, respectively. A filter was not used for the scans. The scans were run through 360° at every 0.25° with 720 ms exposure time for each projection in the male and through 180° at every 0.25° with 720 ms exposure time in the female. Each projection has a pixel size of 2.4 μm in the male and that of 1.7 μm in the female. All projections are 4000 × 2672 pixels in size for both sexes. The resulting X-ray projections were reconstructed using the software NRecon (Bruker micro-CT, Kontich, Belgium). The cross-sections were stacked and processed using the software Amira 6.2.0 (Visualization Sciences Group, Mérignac, France).

### Quantification of female genital damage

Genitalia were cut off, the genital chambers were opened at the lateral side using fine tweezers and pinned on cleaning sponges, and photographs were taken using a Leica M205 A stereomicroscope equipped with a Leica DFC420 camera and the software LAS 3.8 (Leica Microscopy GmbH, Wetzlar, Germany). Outlines of the genital chambers and wounds were traced using the software Adobe Illustrator CS4 (Adobe Inc., CA, USA). Colours were assigned to the scabs (black), scratched areas (dark grey) and genital chamber (light grey). Based on the resulting images, the areas of the genital chambers and wounds were measured using the software Fiji (Schindelin et al. 2012), and the basic statistics were calculated using the software R (R Core Team 2019).

### Analyses of material distribution in subgenital plates and genital chambers

To analyse material distributions in subgenital plates and genital chambers, we applied a confocal laser scanning microscopy-based method established by Michels and Gorb (2012). By applying their method visualising autofluorescence of arthropod cuticles, we investigated the presence of material heterogeneity and interpreted relative stiffness of the relevant structures. This method has been cross-validated by utilising an AFM-based nanoindentation for adhesive setae of a ladybug (Peisker et al. 2013).

Following Michels and Gorb (2012), we prepared samples and recorded micrographs. For this experiment, four males and three females of *M. ornatus* and three males and two females of *P. v. veluchianus* were used. The structures were dissected out in 70% ethanol, then rinsed in 70% ethanol twice and in glycerin twice (≥ 99.5%, free of water, Carl Roth GmbH & Co. KG, Karlsruhe, Germany) and embedded in glycerin on glass slides. Subsequently, we visualised autofluorescence using a Zeiss LSM 700 confocal laser scanning microscope (CLSM) (Carl Zeiss Microscopy GmbH, Jena, Germany) equipped with four stable solid-state lasers with wavelengths of 405 nm, 488 nm, 555 nm and 639 nm. To selectively detect emitted autofluorescence for each of the four laser wavelengths, we applied respectively either a bandpass emission filter transmitting light with wavelengths 420–480 nm or long-pass emission filters transmitting light with wavelengths ≥ 490 nm, ≥ 560 nm or ≥ 640 nm. Objective lenses with × 5 (Zeiss Plan-Apochromat, numerical aperture (NA) = 0.16) and × 10 (Zeiss EC Plan-Neofluar, NA = 0.45) magnification were used. To obtain overview micrographs of each structure, tile scans were performed. To each image produced by the abovementioned four sets of lasers and filters, we assigned blue, green, red (50% saturation) and red (50% saturation). The resulting overlaid micrographs were interpreted according to the colour code reported by Michels and Gorb (2012) as follows: (1) areas coloured with red are likely well-sclerotised and relatively stiff, (2) yellow to green coloured areas are less stiff due to a small amount of resilin presence, and (3) areas visualised with blue are rubber-like protein, i.e. resilin, enriched.

### Finite element simulation of the mechanical performance of the subgenital plate

**Fig. 2:**
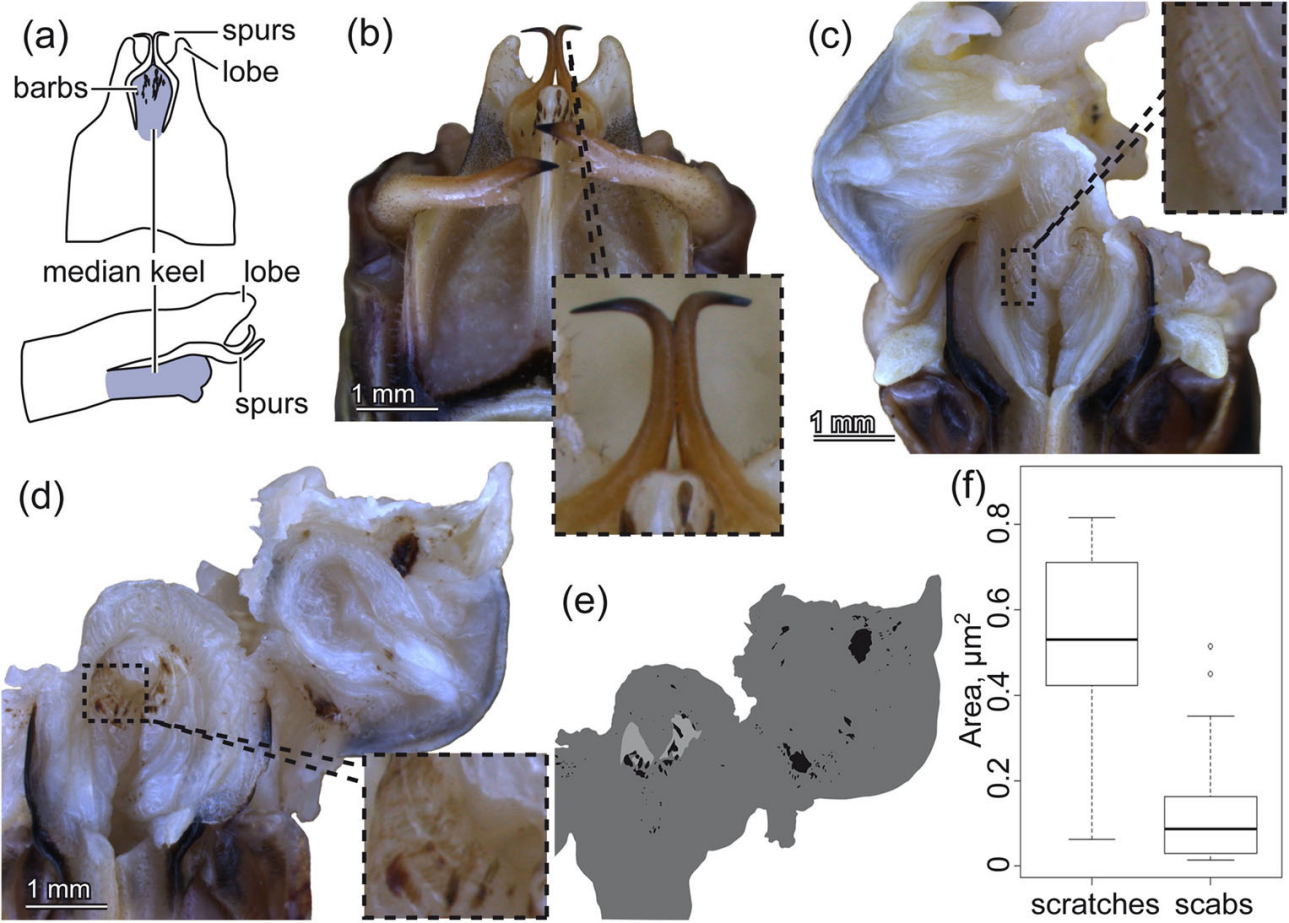
Genital damage in *Metaplastes ornatus*. (**a**) Schemas of the male subgenital plate in the dorsal view (upper) and in the lateral view (lower). (**b**) The subgenital plate from the dorsal side. An inset at the lower right shows the enlarged spurs. (**c**–**e**) Female genital chamber surfaces. Genital chambers were cut-opened (**c**, **d**), outlines of genital chambers (dark grey), scabs (black) and scratched areas (light grey) were traced (**e**), and their sizes were measured (**f**). (**f**) The areas of the measured scratched regions and scabs. The upper whisker (the third quartile plus 1.5 times the interquartile range), the third quartile, the median, the first quartile and the lower whisker (the first quartile minus 1.5 times the interquartile range) were shown from top to down. Outliers are plotted as points

The finite element software package ABAQUS/Standard v6.14 (Simulia, Providence, RI, USA) was used to develop a geometric model of the subgenital plate of *M. ornatus* males. The model, called here as the ‘reference model’, was two-dimensional (Fig. 4) and had dimensions equal to those of the real subgenital plate (Fig. 2b). Based on the CLSM data, we designed our model to consist of three homogeneous parts with different material properties (Fig. 4a). The parts painted in light grey, dark grey and black in Fig. 4a were assumed to have material properties corresponding to resilin-rich cuticle, less sclerotised cuticle and highly sclerotised cuticle and with their elastic modulus set to be 700 MPa, 1.5 GPa and 7.5 GPa, respectively (Rajabi et al. 2017; Eshghi et al. 2018). Taking into account that the Poisson’s ratio of insect cuticle is not yet known, here we assumed it to be 0.3. This is equal to that of many other biological materials, such as bone, and has frequently been used in the literature for simulation of the mechanical behaviour of insect cuticle (Wainwright et al. 1982; Rajabi et al. 2016a, 2017; Wang et al. 2018).

To address the question of how the male reduces risks of damage of the fine spurs during the SRB, we focused on and simulated movements that may cause spur breakage. Considering that the subgenital plate itself is a symmetric and relatively flat plate, we expect that 2D models provide sufficient data for our purpose. Therefore, to reduce the computational time, here we developed a 2D model of the subgenital plate. We cut the model in half about the central line, and a symmetric boundary condition was applied to this symmetric axis (empty triangles in Fig. 4b). Loadings and boundary conditions were applied in three consecutive steps, to simulate the three key steps of hook, pull and push after penetration (Fig. 4b). First, to simulate spurs hooking, the displacements and rotations at the bottom of the model were constrained in all directions and about all axes, respectively (filled triangles in Fig. 4b). The spurs were then rotated counterclockwise by 20°. Second, to simulate the pulling process, the spurs were fixed, and the bottom of the subgenital plate was pulled by applying a displacement of 160 μm. Third, to simulate the pushing process, the spurs were fixed, and the bottom of the plate was pushed by applying a displacement of 100 μm.

To investigate the effect of the specific material composition of the subgenital plate, we developed a second model, called here the ‘stiff model’. In this model, we removed the less sclerotised cuticle and resilin-rich cuticle from the ‘reference model’ and replaced them with the highly sclerotised cuticle (Fig. 4a). For the other comparative case, it is theoretically possible to use a ‘soft model’. However, given that the subgenital plate has to be repeatedly inserted in the narrow space of the female genital chamber, the subgenital plate has to be stiff enough to withstand external forces applied from the female genital chamber not to collapse. Therefore, this obviously malfunctioning case was omitted from further analyses.

Both the models were meshed using the eight-node plane stress elements (CPS8R), which are general-purpose elements. The simulations were performed using a general ‘static step’ (ABAQUS 2007). Each simulation was followed by a mesh convergence analysis to obtain results that are independent of the element size. The von Mises stress criterion was used to examine the risk of the failure of our models under applied loads. According to this criterion, failure occurs when the stress in any element of the model reaches or exceeds the von Mises stress. This is the stress required to distort an element just enough to initiate the yield.

## Results

### Simulated genital coupling

To simulate a genital coupling of *M. ornatus*, we carefully checked the outline of the intact female genital chamber in the volume rendering of the micro-CT scanned female and aligned the subgenital plate on it with the same size-scale (Fig. 1). This simulated genital coupling clearly showed that the height of the subgenital plate is much taller than the intact genital chamber. In addition, the micro-CT images showed that the subgenital plate is a flattened chamber filled, plausibly, with haemolymph. This means that during penetration of the subgenital plate, the genital chamber flattens and deforms the male structure, while the female genital chamber is also deformed. We observed that when we manually pressed the median keel under a stereomicroscope, the radially oriented spurs crossed on the median line.

### Quantification of genital damage

Genital damage was observed in field-caught males and females of *M. ornatus*. Male genital damage was minor. In only four of 23 investigated males, a tip of a spur was broken (e.g. Fig. 2b, ESM 1). By contrast, all of the 23 investigated females carried wounds (Fig. 2b–e, ESM 1). Although it was not always explicit, we could recognise two types of wounds in the genital chamber surfaces. One type was characterised by multiple scratches, and the other one was by scab-like brown patches (Fig. 2c, d). Since the contrast of these wound and background surface colours were not high enough to measure those areas automatically, we marked manually scratched areas and scabs with light grey and black, respectively (Fig. 2e, ESM 1). The areas of the scratched sites and scabs are 0.549 ± 0.189 mm^2^ and 0.138 ± 0.140 mm^2^, respectively. These areas correspond to on average 3.28% and 0.78% of the genital chamber surface area, respectively. Although the proportion of the total genital chamber surface affected is rather small, some wounds were severe (e.g. Fig. 2d). The degree of damage varied between individuals (Fig. 2c–f). The scratches tended to be mainly distributed in the discoidal patch situated on the dorsal side of the genital chamber, and scabs were observed everywhere in the genital chamber (ESM 1).

### Analyses of material distribution in subgenital plates and genital chambers

**Fig. 3:**
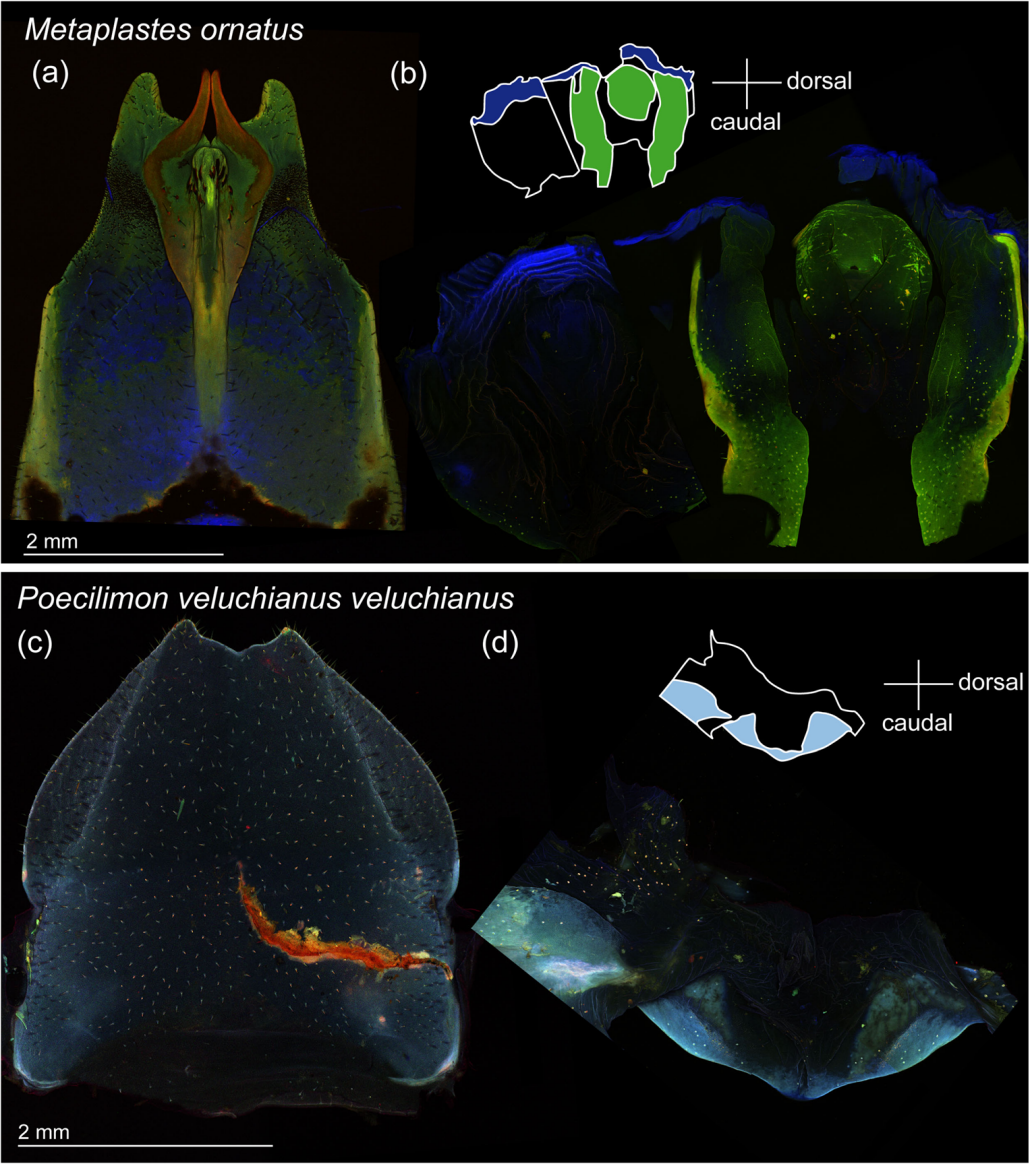
Confocal laser scanning microscopic images of the subgenital plate (**a**, **c**) and genital chamber (**b**, **d**) in *Metaplastes ornatus* and *Poecilimon veluchianus veluchianus*. (**a**, **c**) The ventral view. (**b**, **d**) The genital chamber was opened, and its orientation is shown as the schemes. Each image was taken with different settings so that we cannot directly compare the colour among them, but rather compare variation in material composition within each image. The reddish stripe in the image (**c**) is a wound, and the intensities of the lasers were adjusted by ignoring any signals from the wound

Completely different autofluorescence distributions were observed in the genital structures between the two species, *M. ornatus* and *P. v. veluchianus* (Fig. 3, ESM 1). The subgenital plate and genital chamber of *P. v. veluchianus* were almost evenly coloured with light blue (Fig. 3, ESM 1), indicating that these structures seem to be weakly sclerotised. By contrast, the subgenital plate and genital chamber of *M. ornatus* showed striking material heterogeneity (Fig. 3a, b, EMS 1). From the middle to the basal part, the subgenital plate was dominated by blue colouration, where likely a large amount of resilin is situated, except for the lateral ridges, which are plausibly weakly sclerotised (yellowish green) (Fig. 3a). The middle to the apical part of the subgenital plate features a gable roof-like shape (Fig. 1). This entire region is relatively well-sclerotised with the slightly elevated area on the middle line and the spurs being strongly sclerotised (red) (Fig. 3a). The strong sclerotisation of the spurs continues to the middle of the subgenital plate, resembling S-shaped stripes. The apical region of the spurs is well-melanised, and autofluorescence was hardly detectable (Fig. 3a). The dorsal part of the female genital chamber in *M. ornatus* is less stiff (green) but has sclerotised ridges (yellow) (Fig. 3b). In the apical to the ventral sides, an extremely high intensity of blue was detected, which indicates that these areas are enriched with resilin (Fig. 3b).

### Finite element simulation of the mechanical performance of the subgenital plate

**Fig. 4:**
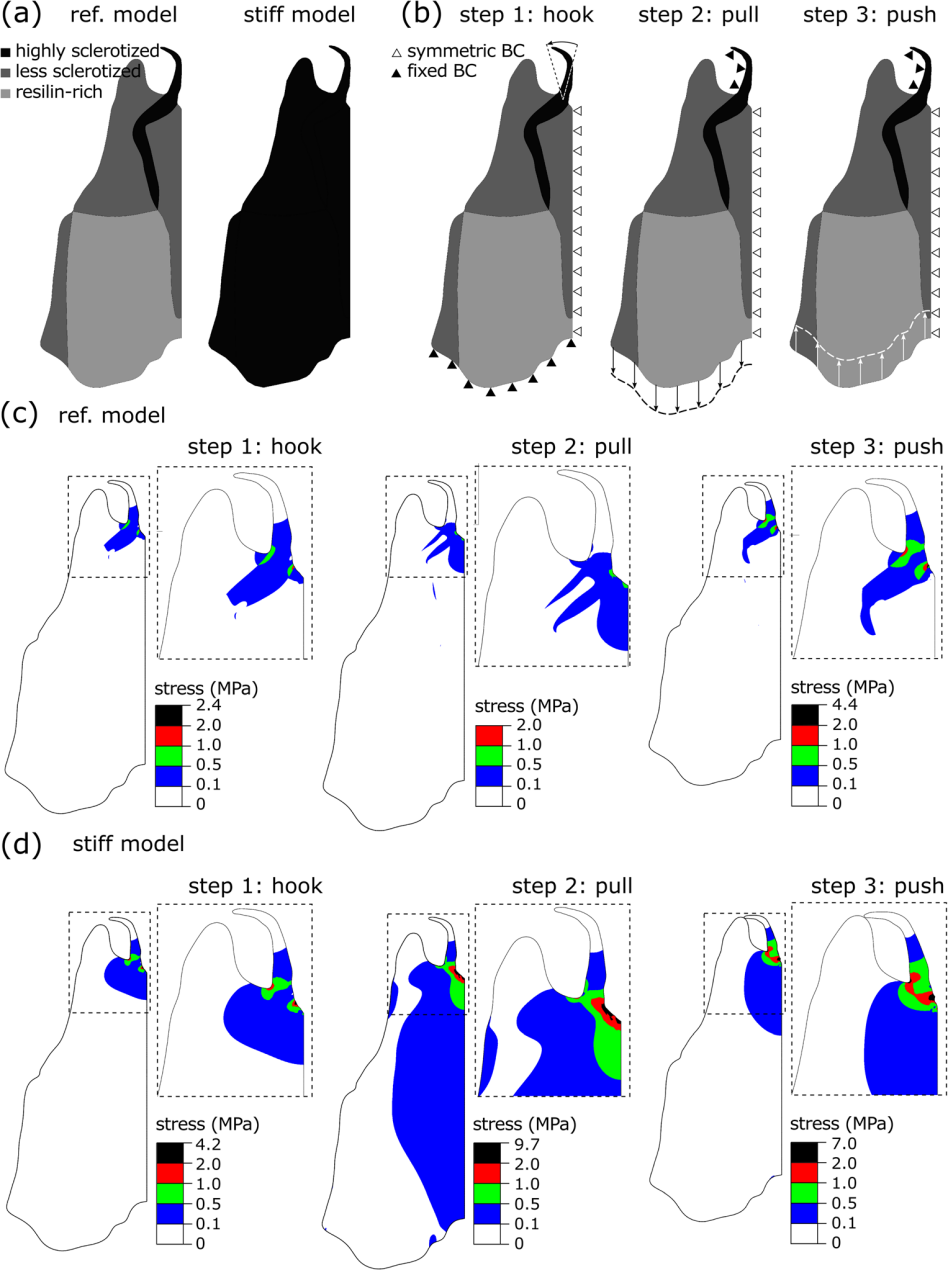
Finite element simulation of the deformation of and stress distribution in the subgenital plate. (**a**) Developed models, including the ‘reference model’ (left) and the ‘stiff model’ (right). The light grey, dark grey and black regions show the parts, which are composed of the resilin-rich, less sclerotised and highly sclerotised cuticle. The models were assumed to be symmetric about the centre line. (**b**) Loadings and boundary conditions for the simulation of the three key steps after penetration: spurs hooking, pulling and pushing. The arrows show the direction of the applied rotations and displacements. Black and white triangles show the fixed and symmetric boundary conditions. (**c**) The distribution of the von Mises stress in the ‘reference model’. (**d**) The distribution of the von Mises stress in the ‘stiff model’. ref., reference; BC, boundary condition

Figure 4 c presents the distribution of the von Mises stress in the subgenital plate of *M. ornatus* (i.e. ‘reference model’). The results were given for the three steps of the spur hooking, pulling and pushing of the subgenital plate. As seen in Fig. 4c, the stress was mainly concentrated at the base of the spur. The maximum stress was equal to 2.4 MPa during the hooking of spurs, reduced during pulling to 2 MPa and reached its maximal value of 4.4 MPa during pushing.

The results obtained from the ‘stiff model’ are shown in Fig. 4d. Here again, the maximum stress occurred at the base of the spurs. However, a different trend from that seen in the ‘reference model’ was found here: the stress was the maximum in pulling, 9.7 MPa, and minimum in hooking, 7 MPa. Comparing the results with those from the ‘reference model’, stiffening the subgenital plate as in the ‘stiff model’ notably changed the distribution and level of the stress. Specifically, the stress in the ‘stiff model’ was not only distributed over a wider area but was also notably higher than that in the ‘reference model’: 1.75 times in hooking, 4.85 times in pulling and 1.6 times in pushing. Electronic supplementary material videos 1 and 2 (ESM 2, 3) show the development of the stress during hooking, pulling and pushing within the ‘reference model’ and the ‘stiff model’, respectively.


ESM 2(MP4 5048 kb)ESM 3(MP4 5586 kb)

## Discussion

This study addresses for the first time in *M. ornatus* the biomechanics of a male genital structure involved in SRB and how they are adapted to reduce mechanical damage of the fine spur. Our experiment of genital coupling simulated using a micro-CT-based visualisation technique suggested that the depth of the subgenital plate is much greater than that of the female genital opening at rest in *M. ornatus*. Although females slightly open their genital chamber just before males start the SRB (see a supplementary video in Foraita et al. 2017), the subgenital plate must be more or less flattened, when this structure is inserted in the narrow female genital chamber. This movement likely causes the paired spurs to move in a way that they cross each other medially, as we observed under a stereomicroscope, which presumably assists the subgenital plate with penetrating.

While males perform back-and-forth movements of their subgenital plate within the female genital chamber, the two pointed spurs and sharp barbs on the median keel have to interact with the genial chamber surface due to its narrow space. This could explain why many wounds were found on the genital chamber surfaces of all of the female individuals caught in the field. In the genital chamber, a resilin-enriched region observed only in *M. ornatus* (i.e. not in *P. v. veluchianus*, a species where the SRB is absent) presumably reduces the risk of severe mechanical damage and increases female tolerance. This is thus functionally equivalent to the resilin-rich composition of the spermalege in female bed bugs, *Cimex lectularius*, which likely functions in avoiding damage at the site of hypodermic traumatic insemination (Michels et al. 2015). The resilin-enriched region in *M. ornatus* lies at the most apical region on the genital chamber, where it should function to withstand impacts when males vigorously insert the subgenital plate. Resilin is an elastomeric protein, which is known for its remarkable resilience (Michels et al. 2016). Under loading, the soft resilin-rich areas deform and thereby reduce the stress level on other areas. The presence of resilin is, in general, a widespread adaptation to minimise mechanical damage as known, for example, for the wings of Pterygota (Wootton et al. 2003; Mountcastle and Combes 2014; Rajabi et al. 2016b; Rudolf et al. 2019) and the slender tibiae of stick insects (Schmitt et al. 2018). A mechanical adaptation of the female reproductive tract is recently reported also in a vertebrate, the common bottlenose dolphin, *Tursiops truncatus* (Orbach et al. 2019).

In contrast to the widespread genital damage observed in females, only a few field-caught males exhibited a broken tip of the two spurs (ca. 17%). Sperm removal ability is essential for *M. ornatus* males. The sperm removal efficiency is relatively high, implying weak sperm competition levels, and likely as an evolutionary consequence, relatively small testes were detected in *M. ornatus* (Winkler et al. 2019). The two spurs and sharp barbs on the median keel seemingly play an important role to keep the subgenital plate in the female genital chamber during intercourse, since female behaviour can often involve significant resistance to male SRB attempts (YM, SAR & KR pers. observ. 2019).

If a function of the spurs and sharp barbs is solely to hook the genital chamber tightly, the spurs would have to point backwards to hook the genital chamber surface. However, none of the spurs is pointed or bent backwards (ESM 1). Indeed, for repeated use of the subgenital plate, males must be able to easily unfasten the spurs from the genital chamber surface as well. The spurs’ shape and stiffness enable them to slide along the genital chamber surface without its deformation and breakage. Moreover, we found a marked material heterogeneity in *M. ornatus* subgenital plates that contrasted strongly with the homogeneously and weakly sclerotised subgenital plate of *P. v. veluchianus*, which do not show the SRB. It is reasonable to expect that males cannot even insert the *P. v. veluchianus* type of the subgenital plate since such a subgenital plate would collapse due to external forces generated by the narrow female genital chamber. Comparative finite element modelling comparing models with the realistic material heterogeneity versus a homogeneously stiff material demonstrated that in all simulated loadings, the subgenital plate with the material heterogeneity experiences lesser stresses than the homogeneous model. Considering that the number of thrusts (i.e. loading cycles on the subgenital plate) in a single mating can exceed 2000 (Foraita et al. 2017), our results suggest that the specific material heterogeneity of the subgenital plate of *M. ornatus* plays an important function to reduce the stress concentrations within the structure and thereby increases its durability. In addition to the material heterogeneity, we expect the specific shape of the sclerotised stripes to play a role in preventing high-stress concentrations at the base of the spurs. However, a detailed investigation of its contribution to the biomechanics of the SRB remains a question for future investigation.

Our finite element results suggested that, during the SRB, the base of the spurs experiences a stress higher than other regions. Therefore, it seemingly contradicts our observation that all of the cases of natural spur breakage occurred at the tip of the spur. One reason for this discrepancy between the numerical results and the observations may be that the spurs are not fully homogenous, as was considered in our models. Due to the strong melanisation of the apical region of the spur, autofluorescence was hardly detected. Material properties of the entire spur need to be measured using alternative methods, e.g. nanoindentation. Besides, the loadings assumed in our simulations may not exactly replicate those acting on the spurs in practice. We assumed that, during the SRB, the loads are distributed uniformly along each spur (steps 2 and 3, Fig. 4b). However, this might not always be the case. We performed additional numerical simulations to understand how this can influence the stress developed in the spurs. We found that applying the loads closer to the spur tip shifts the maximum stress towards where most of the breakages took place (ESM 1). The desideratum of further studies is to visualise exact interactions between the subgenital plate and vagina, for instance using the synchrotron-based *in vivo* X-ray cineradiography as done for another bushcricket species *Metrioptera roeselii* (Wulff et al. 2017), so that we can simulate the behaviour of the subgenital plate precisely. Our study provides a platform for future more realistic modelling of the complicated SRB.

## Conclusion

Our study demonstrated that the observed material heterogeneity of the subgenital plate and genital chamber surface play roles for reducing genital damage for both sexes in *M. ornatus*. This is likely an adaptation to the SRB in the species. Since the mechanical aspect has not previously been investigated in the context of SRB, our findings open up a new perspective on understanding the evolution of this behaviour and male genital structures.

## Supplementary information

ESM 1 (PDF 4456 kb)

## Data Availability

All supporting data are available as supplementary materials except for micro-CT data, which are available from the corresponding author upon reasonable request.
