# Supplementary material for: Material heterogeneity of male genitalia reduces genital damage in a bushcricket during sperm removal behaviour
Source: Naturwissenschaften. 2020 Nov 25;107(6):52. doi: 10.1007/s00114-020-01706-w (PMC7688094; doi:10.1007/s00114-020-01706-w)
Supplement: Supplementary file 3 — (PDF 4456 kb) [file 114_2020_1706_MOESM1_ESM.pdf]

Electronic Supplementary Material 1 (Fig. S1-S7)

Material heterogeneity of male genitalia reduces genital damage in a bushcricket during sperm removal behaviour

Yoko Matsumura[1*], Mohsen Jafarpour[1], Steven A. Ramm[2], Klaus Reinhold[2], Stanislav N. Gorb[1], Hamed Rajabi[1]

[1] Department of Functional Morphology and Biomechanics, Zoological Institute, Kiel University, Am Botanischen Garten 1–9, 24118 Kiel, Germany

[2] Department of Evolutionary Biology, Bielefeld University, Konsequenz 45, 33615 Bielefeld, Germany

[*]yoko.matumura.hamupeni@gmail.com

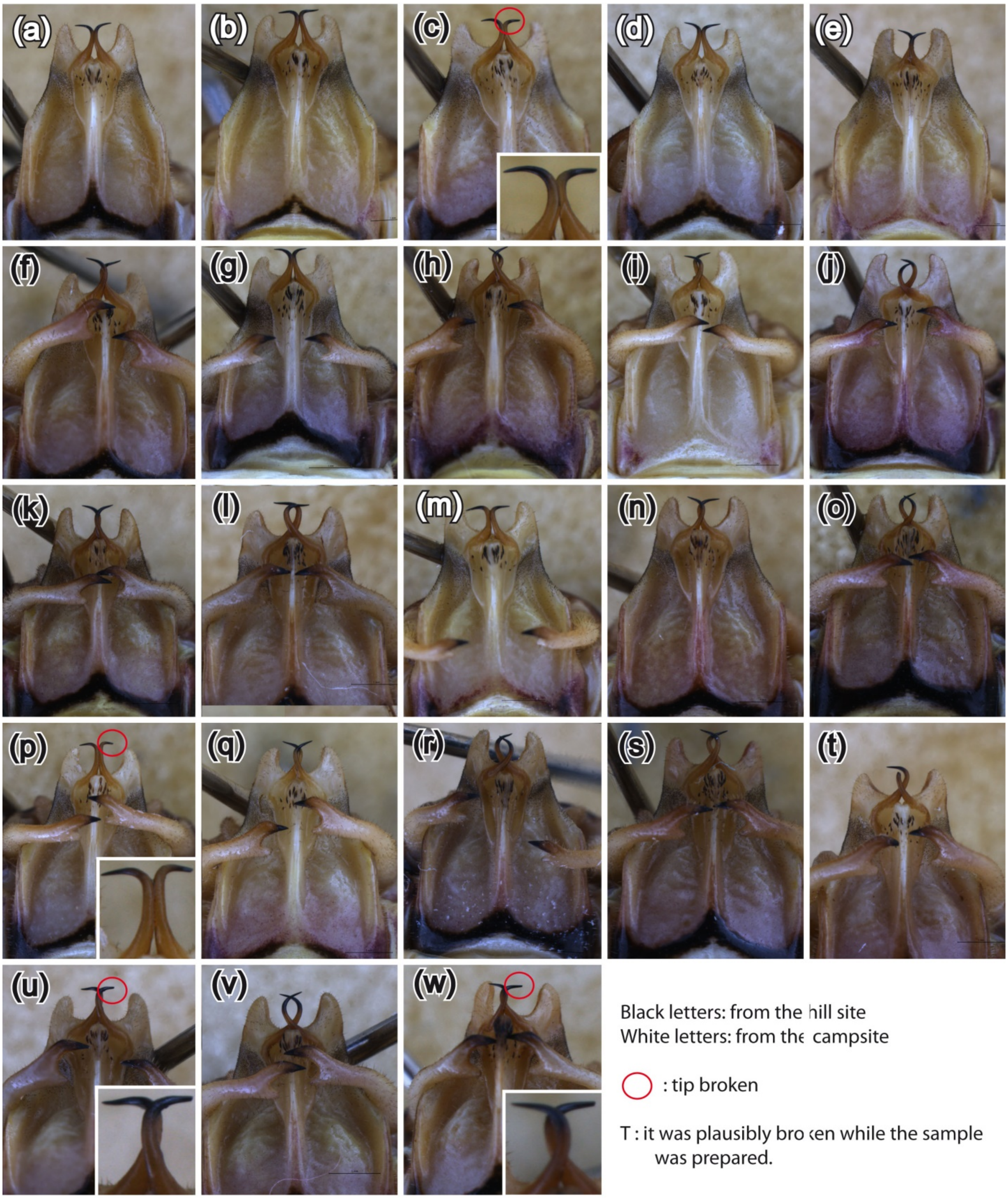

Fig. S1. Genital damage in 23 individual males of *Metaplastes ornatus*. The colour differences in the labels represent their collection sites, i.e. black ones in Paleokastro, central Greece (Nomos Fthiothis), and white ones were collected a few km north of the village of Vitoli, central Greece.

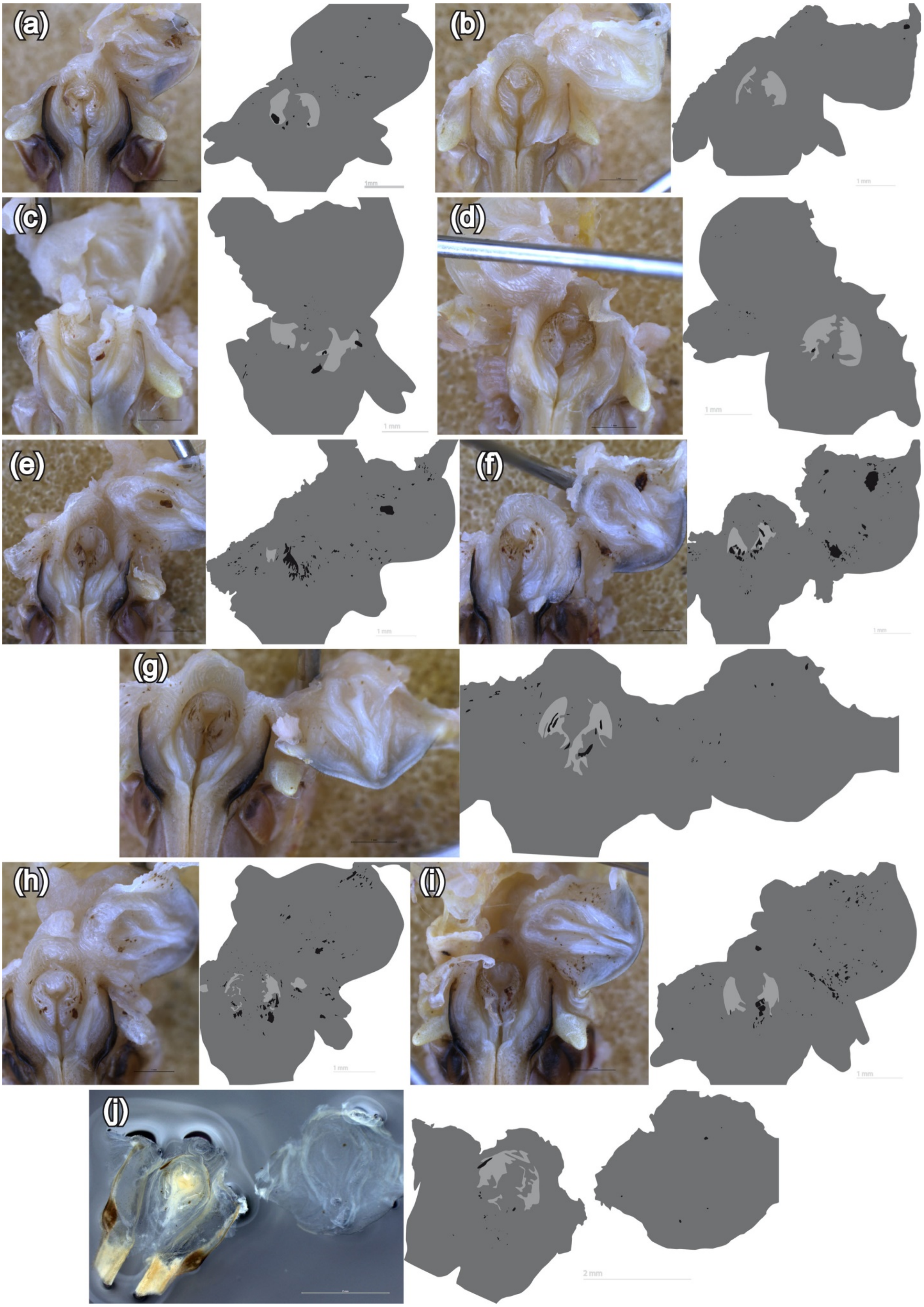
(a)
1mm
(b)
1 mm
(c)
1 mm
(d)
1 mm
(e)
1 mm
(f)
1 mm
(g)
(h)
1 mm
(i)
1 mm
(j)
2 mm

Fig. S2. Genital damage in 23 individual females of *Metaplastes ornatus*. The colour differences in the labels represent their collection sites, i.e. black ones in Paleokastro, central Greece (Nomos Fthiothis), and white ones were collected a few km north of the village of Vitoli, central Greece.

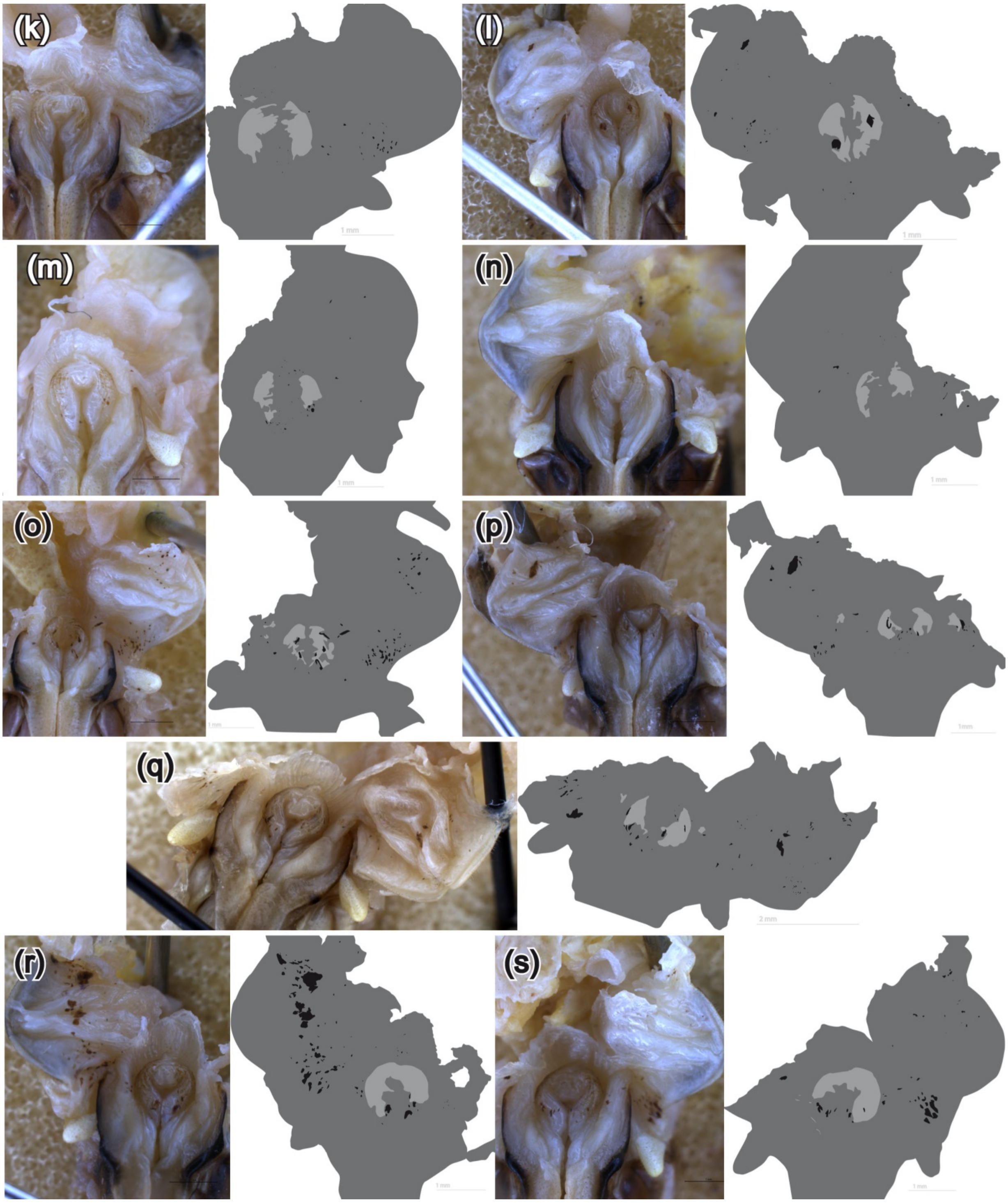

Fig. S2. (cont.)

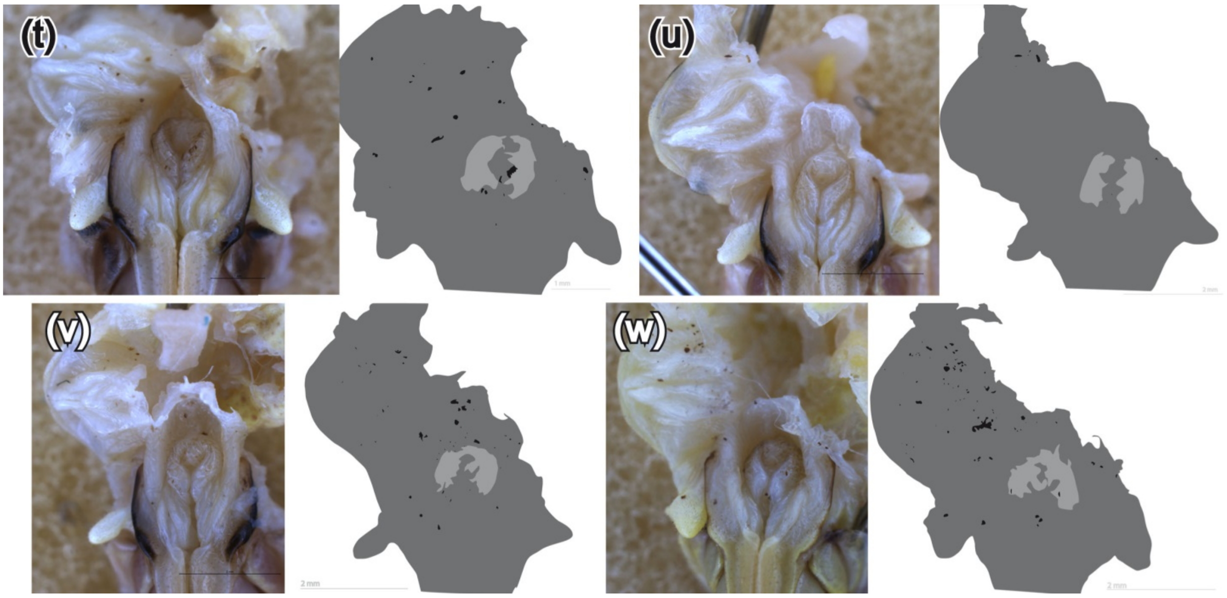

Fig. S2. (cont.)

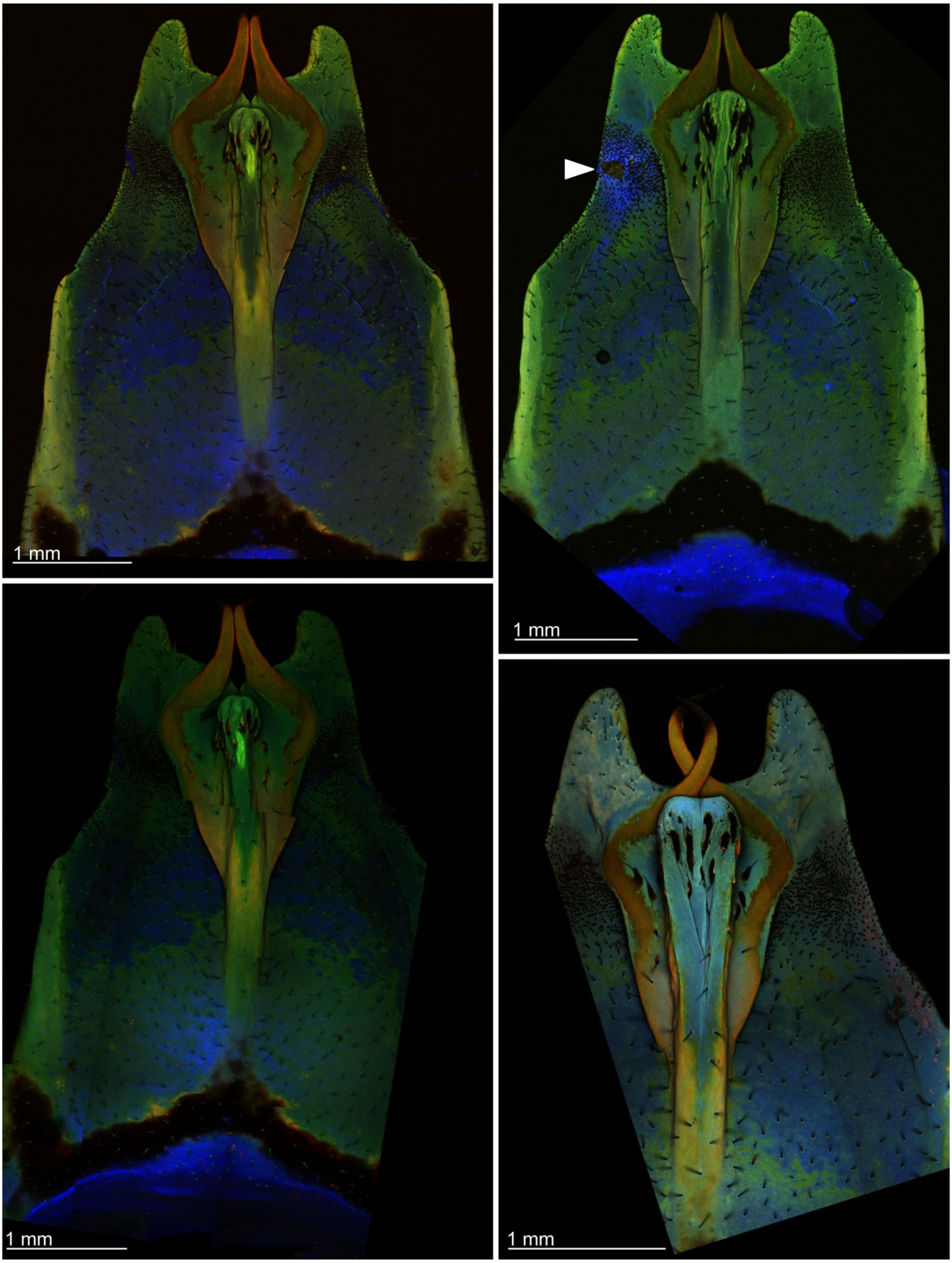

Fig. S3. Confocal laser scanning microscopic images of the subgenital plate in *Metaplastes ornatus.*

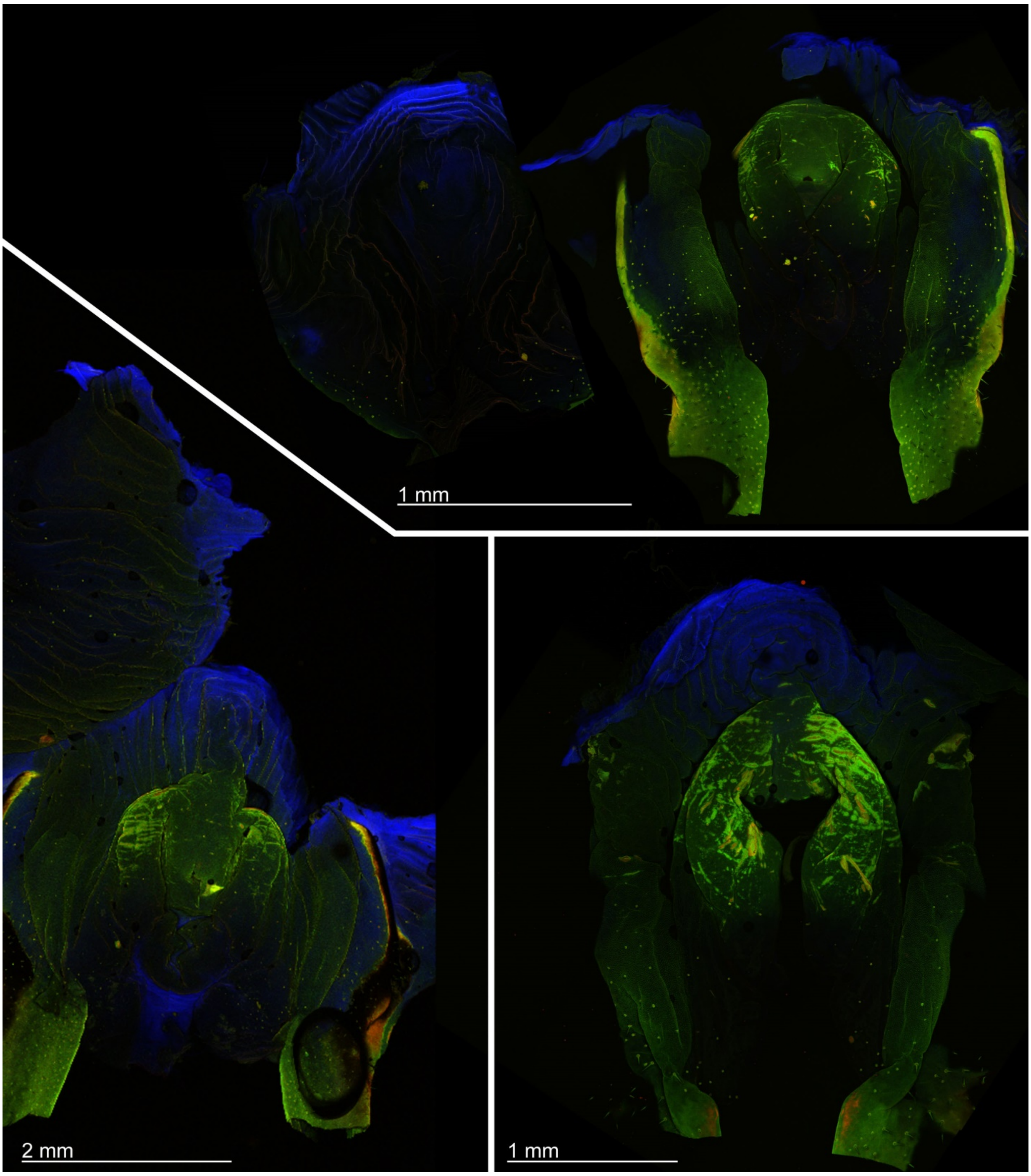

Fig. S4. Confocal laser scanning microscopic images of the genital chamber in *Metaplastes ornatus.*

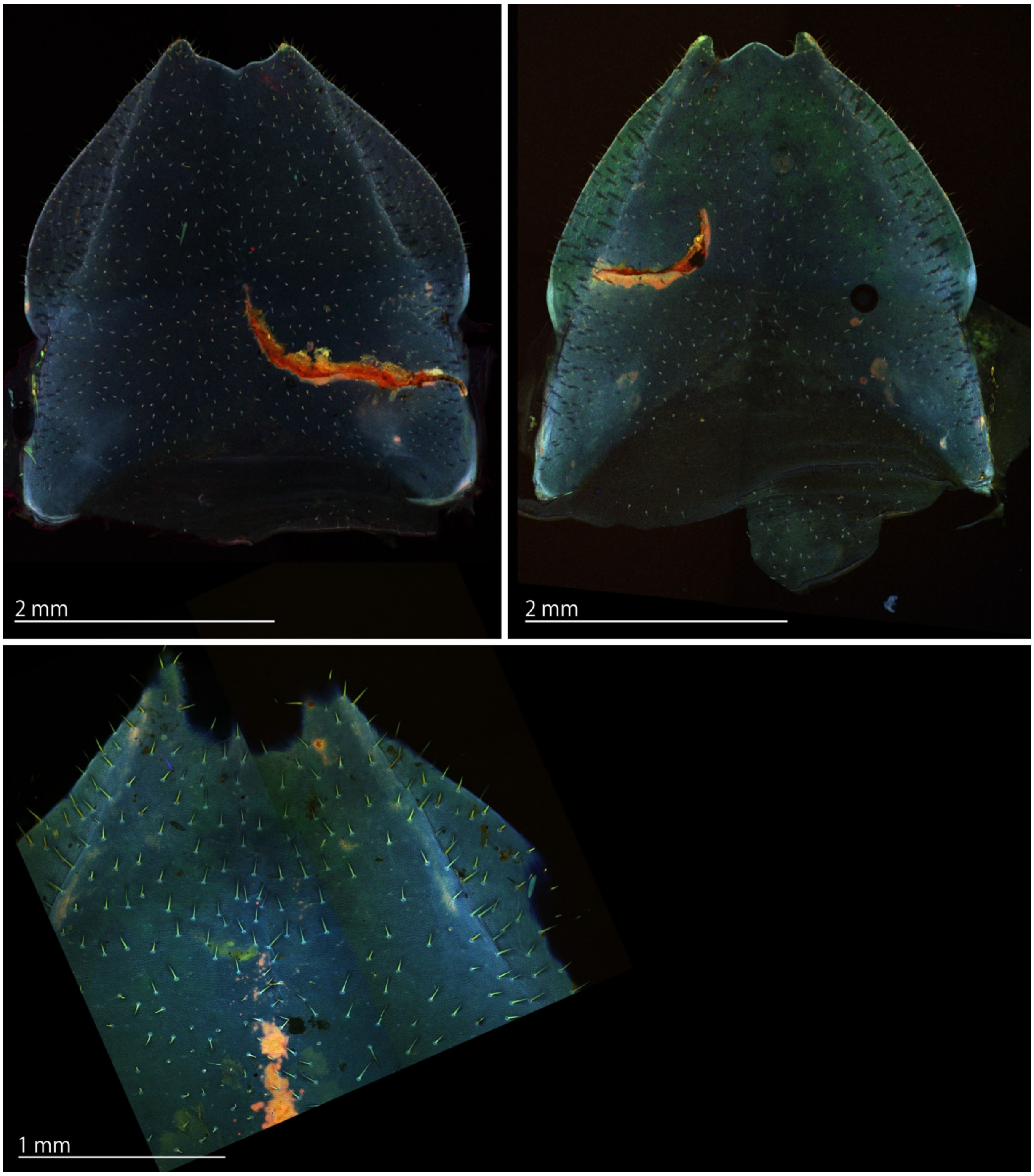

Fig. S5. Confocal laser scanning microscopic images of the subgenital plate in *Poecilimon veluchianus veluchianus*.

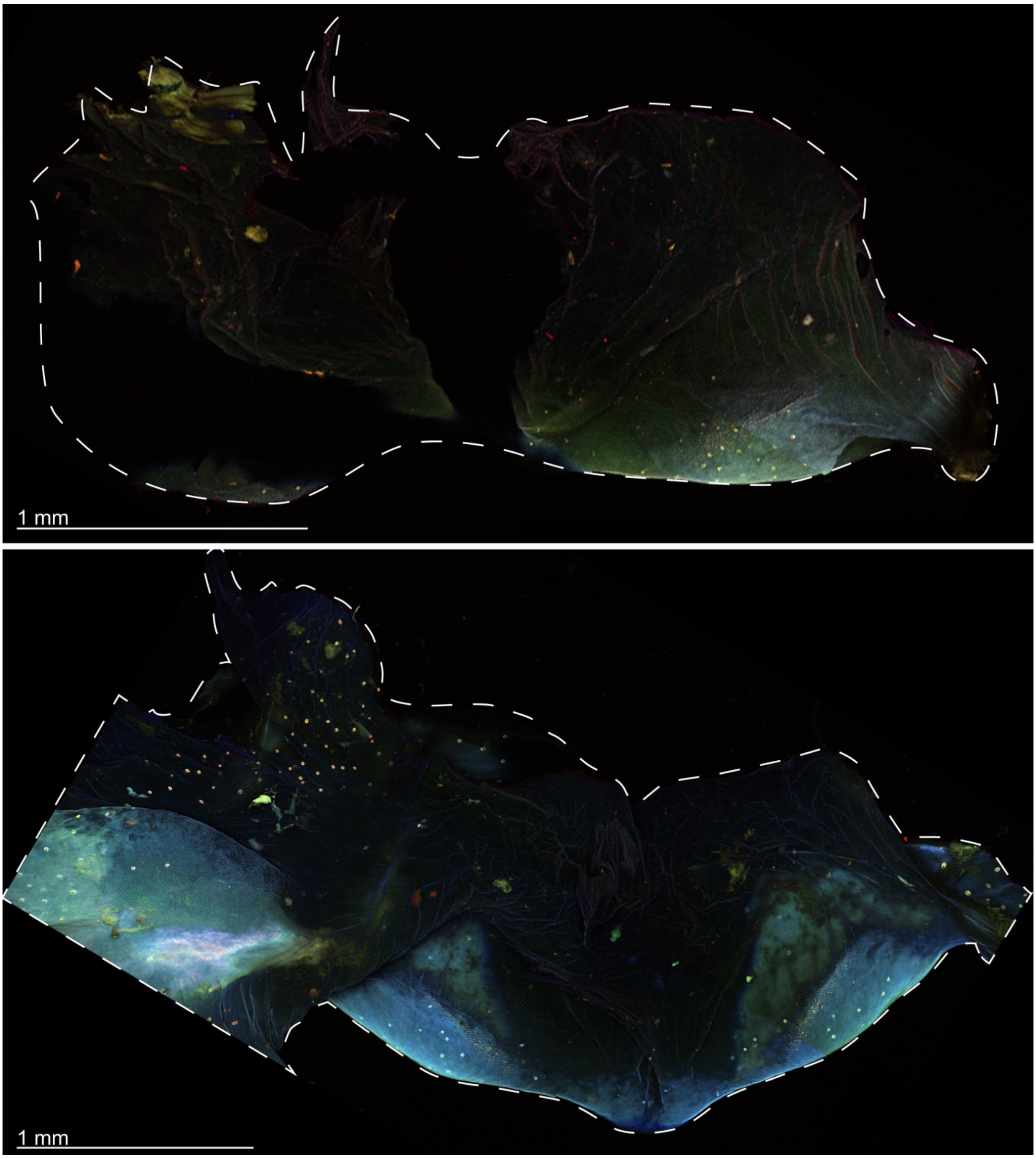

Fig. S6. Confocal laser scanning microscopic images of the genital chamber in *Poecilimon veluchianus veluchianus*.

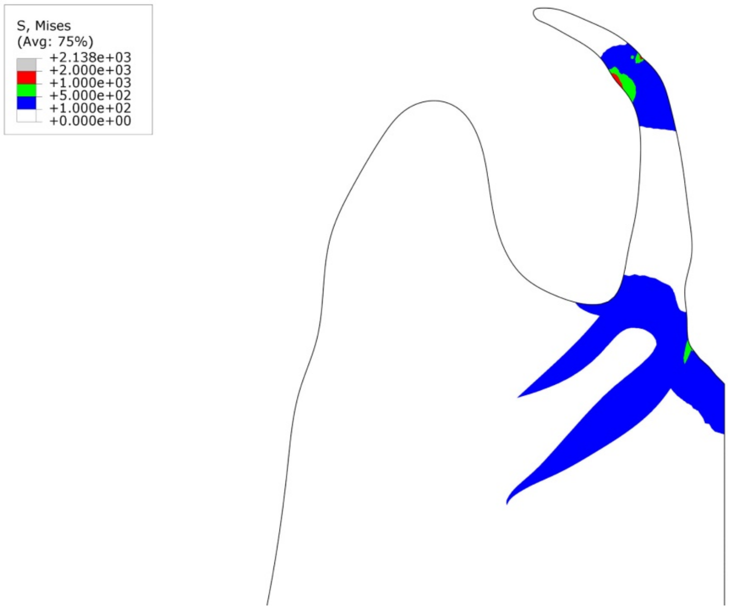

Fig. S7. The distribution of the von Mises stress in the 'reference model'. The model was assumed to be symmetric about the centre line. One of the three key steps after penetration, i.e., pulling, was simulated. Loading and boundary conditions were the same as that of the simulation in the main text (Fig. 4b step2: pull), except that the load applied to the spur was concentrated at the tip.
